# Association between FDG accumulation in interstitial lesions and acute exacerbation risk in lung cancer: multicenter analysis

**DOI:** 10.1007/s11604-025-01869-4

**Published:** 2025-09-12

**Authors:** Yuriko Ishida, Shiro Watanabe, Jun Sakakibara-Konishi, Yasuyuki Ikezawa, Hajime Kikuchi, Yasutaka Kawai, Hirokazu Kimura, Sho Nakakubo, Kenji Hirata, Kohsuke Kudo, Satoshi Konno

**Affiliations:** 1 Department of Respiratory Medicine, Faculty of Medicine, Hokkaido University, Sapporo, Japan; 2 Department of Diagnostic Imaging, Graduate School of Medicine, Hokkaido University, Kita15, Nishi7, Kita-ku, Sapporo, Hokkaido 060-8638 Japan; 3 Department of Nuclear Medicine, Hokkaido University Hospital, Sapporo, Japan; 4 Department of Respiratory Medicine, Obihiro Kosei Hospital, Obihiro, Japan; 5 Department of Respiratory Medicine, Oji General Hospital, Tomakomai, Japan

**Keywords:** Interstitial pneumonia, Lung cancer, Acute exacerbation, FDG, PET, SUV

## Abstract

**Purpose:**

Interstitial pneumonia (IP) is associated with poor prognosis in lung cancer and increases the risk of acute exacerbation (AE). Few studies analyzed the relationship between fluorodeoxyglucose (FDG) accumulation in IP with lung cancer complicated with IP and the incidence of AE. This study investigates the association between FDG accumulation in the interstitial lesions and the AE incidence in patients with lung cancer complicated with IP.

**Materials and methods:**

This multicenter, retrospective study included patients with lung cancer complicated with IP who received chemotherapy. All CTs at baseline and the onset of AE were centrally adjudicated. The SUVpeak and FDGscore for interstitial lesions were calculated from FDG positron emission tomography images before chemotherapy, and these values were corrected using reference uptake. To determine the association with AE risk, clinical characteristics and imaging findings were compared between patients who developed AE and those who did not. Subsequently, logistic regression analysis was performed to identify risk factors for the development of AE.

**Results:**

One hundred and thirteen patients who met the eligibility criteria were enrolled from three centers. However, 9 patients with a clinical diagnosis of collagen-related interstitial pneumonia were excluded due to predominant FDG accumulation in the interstitial lesions, and 104 patients were analyzed. Of those patients, 31.7% (33/104) developed all grade AE and 18.3% (19/104) developed grade 3 or higher. There were no significant differences in patient characteristics and imaging patterns between those with and without AE. SUVpeak in the ipsilateral and contralateral interstitial lesions to the tumor and the FDGscore did not differ between those with or without AE.

**Conclusions:**

No association was observed between FDG accumulation in interstitial lesions and AE in patients with lung cancer complicated with IP. We may have to remain cautious about the risk of AE in lung cancer complicated with IP, even when FDG accumulation in interstitial lesions is high or low.

**Supplementary Information:**

The online version contains supplementary material available at 10.1007/s11604-025-01869-4.

## Introduction

Interstitial pneumonia (IP) complicates 5–10% of lung cancer patients at diagnosis, and anticancer drug therapy causes acute exacerbations (AE) in 5–20% of patients, 30–50% of whom become fatal [1]. Lung cancer complicated with IP has a high risk of AE by anticancer drug therapy, the available drug options are limited, and the prognosis is poor. Therefore, anticancer drug therapy is a double-edged sword, and careful decisions must be made as to which patients can be given chemotherapy, as well as the identification of risk factors that can cause AE. Some previous studies analyzed several risk factors for that and suggested honeycomb lung [2, 3], forced vital capacity (FVC) [4, 5], etc. While it is important to determine pre-existing honeycomb lung, even among radiologists specializing in IP, it is difficult to differentiate honeycomb lung from emphysema completely, and there is a low concordance rate in identifying honeycomb lung [6]. Regarding FVC, there are also reports that low FVC is associated with AE [7]. However, since many lung cancer patients complicated with IP have a history of smoking and often have emphysema, the FVC of combined pulmonary fibrosis and emphysema may be masked and overestimated due to overinflated emphysema [8, 9]. Therefore, it is difficult for a nonspecialist physician to accurately assess and appropriately use these indices.

[^18^F] fluorodeoxyglucose positron emission tomography (FDG-PET) and computed tomography (CT) are commonly used modalities for lung cancer staging and lesion evaluation. FDG is taken up by glucose transporters (GLUTs) and enters the glycolytic system, where it accumulates. There are about 100 different types of GLUTs, and it has been reported that elevated standardized uptake value (SUV) correlates with GLUT1-positive cell rate in lung cancer [10]. The association between FDG-PET/CT and IP has also been mentioned, suggesting that FDG accumulation in fibrotic areas in the interstitial regions of IP is associated with activity and prognosis [11, 12]. Various mechanisms have been implicated as to why FDG accumulates in IP, including increased expression of GLUT1 and hypoxia-inducible factor (HIF) in inflammatory cells and red blood cells [12] and induction of GLUT1 by mutant growth factor-β (TGF-β), which is involved in fibrosis progression [13].

Several previous studies showed that preoperative FDG accumulation in the interstitial lesion of lung cancer complicated with IP were associated with AE. In another study that evaluated short-term survival in lung cancer complicated with IP after lung resection, there were no AE in the group with SUVmax less than 2.55 [14]. On the other hand, few studies have investigated the FDG accumulation in interstitial region of lung cancer complicated with IP and the risk of chemotherapy-induced AE. One study reported that 7 of 33 patients with lung cancer with interstitial lung disease developed AE and that the background lung FDG accumulation was significantly higher in patients who developed AE [15], but the number of cases in this single-center study was limited. In this multicenter study, we increased the number of cases and examined the association between FDG accumulation in the interstitial lesion and the incidence of AE during chemotherapy in patients with lung cancer complicated with IP.

## Materials and methods

### Ethics approval and consent to participate

This study was conducted in accordance with the Declaration of Helsinki. The study protocol was approved by the Ethics Committee of the Hokkaido University and the Institutional Review Board or Ethics Committee of other participating facilities (#022-0274). According to the Ethical Guidelines for Medical Research on Human Subjects in Japan, this research does not involve intervention or use of samples obtained from the human body. The need for patient consent was waived as this was a retrospective study, and anonymity was secured. Therefore, the Institutional Review Boards or Ethics Committees of all participating facilities approved the use of the opt-out method, publishing the study on either the participating facility’s website or on a bulletin board.

### Study population

This multicenter, retrospective study involved three institutions in Japan and included patients with (1) advanced or recurrent lung cancer, (2) comorbid idiopathic IP, and (3) FDG-PET/CT performed within 3 months of chemotherapy. Exclusion criteria included no IP by central judgment, no PET/CT imaging, no history of anticancer therapy, and no history of chest or mediastinal radiation therapy. Pre-existing IP was classified as usual interstitial pneumonia (UIP), probable UIP, indeterminate for IP, and alternative diagnosis, according to the official American Thoracic Society/European Respiratory Society/Japanese Respiratory Society/Asociacion Latinoamericana del Torax (ATS/ERS/JRS/ALAT) statement (2022) [1]. Connective tissue diseases associated interstitial lung disease (CTD-ILD) is collagen disease-associated interstitial pneumonia. In this study, patients were defined as “those with a diagnosis of CTD-ILD after a visit to a collagen disease physician”, not a case of suspected collagen disease or a diagnosis of “Idiopathic Pulmonary Fibrosis Associated with Features of Autoimmunity”.

### Clinical and laboratory data collected for analysis

Clinical and laboratory data used in this study were retrieved from patient medical records, including age, sex, height, body weight, smoking history, performance status (PS), SpO_2_ at rest, tumor histology, stage at diagnosis (based on the eighth edition of the TNM staging system of lung cancer), programmed cell death ligand 1 (PD-L1) status, the presence of druggable driver mutation, usage of home oxygen therapy, history of acute exacerbation of pre-existing IP, administration of steroids and anti-fibrotic agents, history of surgical resection for lung cancer, history of palliative radiation therapy, blood tests at the administration of chemotherapy and onset of AE (for albumin, C-reactive protein, hemoglobin, Krebs von den Lungen 6, lactate dehydrogenase, presence of anti-nuclear antibody), pulmonary function tests (%FVC, FVC1.0%, and % diffusing capacity for carbon monoxide (%DLco)), the latest date available to identify outcomes, survival outcomes, and cause of death in fatal cases.

### FDG-PET/CT acquisition protocol

Imaging was conducted at each hospital using a total of 5 PET/CT systems including Biograph 64 (Siemens Healthcare GmbH, Erlangen, Germany); Discovery ST, Discovery 610, Discovery MI (GE HealthCare, Milwaukee, WI); and Gemini TF (Philips Healthcare, Cleveland, OH). As part of the clinical protocol, scans were acquired and reconstructed in accordance with institutional standards, following the guidelines established by the Japanese Society of Nuclear Medicine in 2018.

### Evaluation of HRCT at baseline and the onset of AE

All diagnostic chest CTs were centrally adjudicated individually by two respiratory physicians who performed visual assessments without clinical information, and in cases where the assessments differed, a third physician made the final decision. AEs were defined as those that occurred within 6 months of the end of chemotherapy. The onset of AE was diagnosed by attending respiratory physicians according to clinical features and CT findings. Other diseases, such as infection, cancer progression, congestive heart failure, and radiation pneumonia, were carefully excluded. CT findings at the onset of AE were classified as acute interstitial pneumonia (AIP)/diffuse alveolar damage (DAD)-like pattern, hypersensitivity pneumonia (HP)-like pattern, cryptogenic organizing pneumonia (COP)-like pattern, nonspecific interstitial pneumonia (NSIP)-like pattern, and others according to the ATS/ERS international multidisciplinary classification of IP [16].

### Evaluation of FDG-PET at baseline

**Fig. 1:**
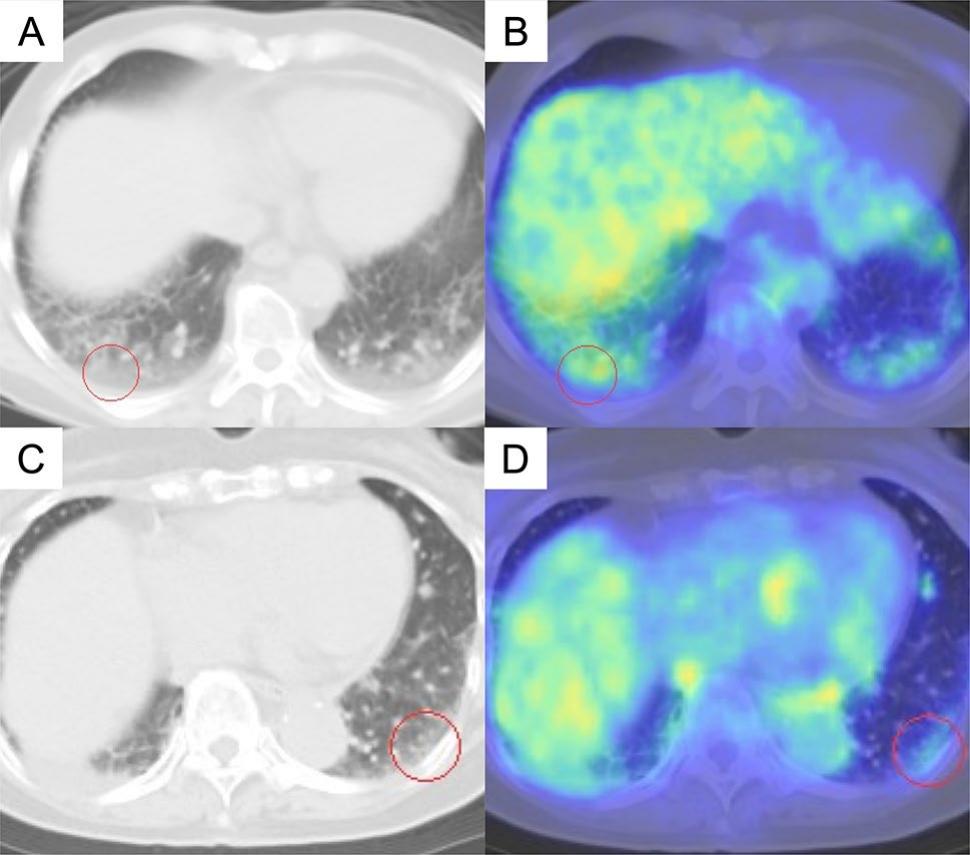
FDG accumulation in the interstitial lesions. Representative cases of strong (**A**, **B**) and weak (**C**, **D**) FDG accumulation with SUVpeak values of 2.30 and 1.72, respectively, on PET-CT in the interstitial lesions

**Fig. 2:**
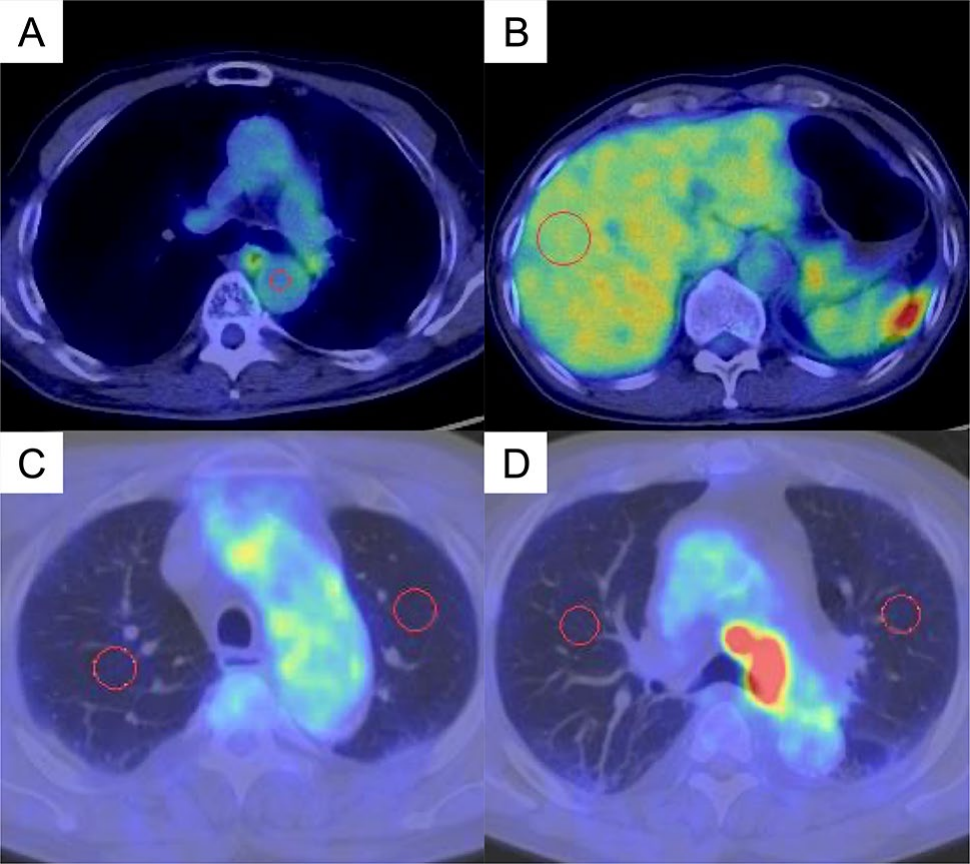
Reference organs to correct for SUVpeak. **A** A spherical volume of interest (VOI) was set at 5 mm for the aorta, **B** 30 mm for the liver, and VOI of the lungs was 20 mm, and the mean values of **C** the aortic arch and **D** tracheal bifurcation of the bilateral lungs were used

FDG-PET/CT images were interpreted independently by a board-certified radiologist and a respiratory physician. The peak standardized uptake value (SUVpeak) of each interstitial lesion, which we identified on the CT images of patients with IP, and the tumor were measured using a spherical region-of-interest with a variable diameter on PET images (Fig. 1). The value of a 1 cm^3^-volume spherical volume of interest (VOI) where FDG uptake centering around the hottest point in the foci was defined as the SUVpeak [17]. Technical and physiological factors may influence SUVs. Therefore, especially in a multicenter trial, it is preferred not to use absolute SUVs but residual uptake values in a tumor area normalized to an intra-image reference uptake. The target-to-blood ratio (TBR) was calculated using a spherical VOI set at 5 mm in the aorta as reference uptake, the target-to-liver ratio (TLR) set at 30 mm in the liver, and the target-to-normal ratio (TNR) set at 20 mm in the ipsilateral and contralateral normal lung in the same slice at the aortic arch and tracheal bifurcation (Fig. 2). The SUVpeak for both bilateral lung interstitial lesions were calculated from FDG-PET/CT images before chemotherapy and included those corrected by dividing by the SUVmean of references. The ipsilateral side refers to the lung with the main recurrent lesion, whereas the contralateral side refers to the other lung.

In this study, FDGscore was evaluated at 20 sites to assess the extent of FDG accumulation in interstitial lesions: the anterior, middle, and posterior regions of both lungs at three anatomical levels (aortic arch, tracheal bifurcation, and maximal right atrial level), and at the diaphragmatic surface. Each site was scored on a 5-point scale: 0, no uptake; 1, uptake below the blood pool; 2, uptake above the blood pool and below the liver; 3, uptake equivalent to more than the liver; and 4, uptake markedly higher than the liver. Sites overlapping with tumors were deemed unevaluable. The total score ranged from 0 to 80 points. FDGscore was defined as the average score of the two evaluators divided by the full score, excluding the unevaluable sites. We used the FDGscore to conduct analyses comparing patients with and without AE.

### Statistical analysis

Categorical variables were presented as numbers (percentages) and compared using Fisher’s exact test. Non-normally distributed continuous variables were presented as median (interquartile ranges) and compared using a Mann–Whitney *U* test. A *p* value of < 0.05 was considered statistically significant. First, in order to investigate the relationship between clinical or imaging factors and FDG accumulation in interstitial lesion, we divided the patients into two groups based on the presence or absence of nominal variable factors and the median of continuous variable factors, and compared SUVpeak. Next, to investigate the association of various parameters with the risk of AE, clinical characteristics, baseline HRCT findings, FDG-PET/CT parameters, and various laboratory data were compared between patients who developed AE and those who did not. Subsequently, logistic regression analysis was performed to identify risk factors for the development of AE.

## Results

### Patient characteristics

**Fig. 3:**
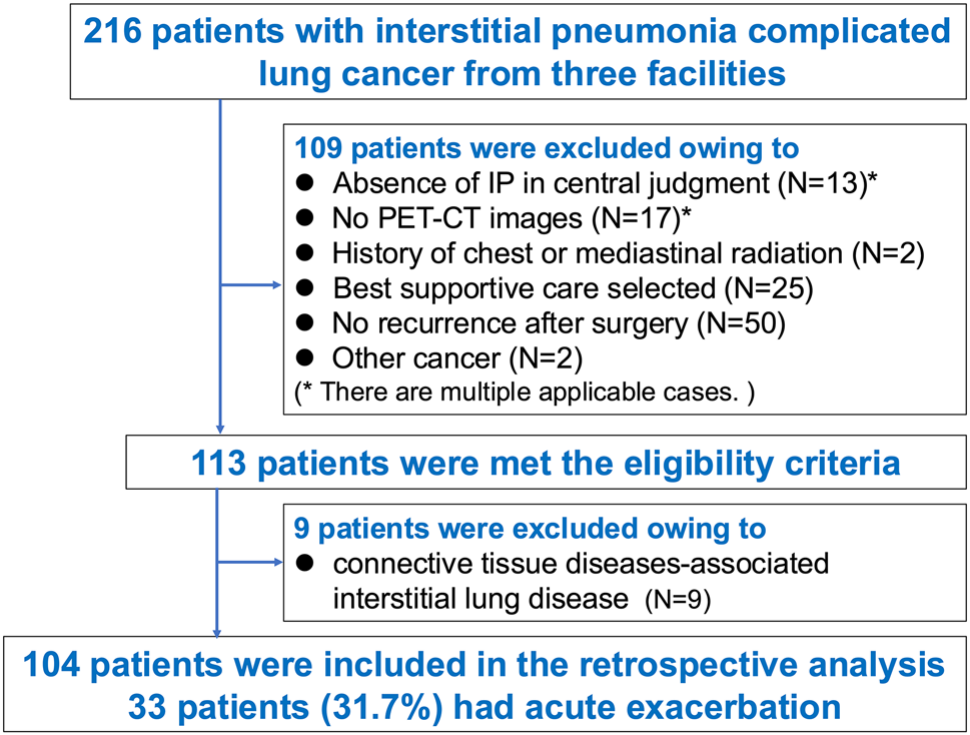
Patient disposition. *IP* interstitial pneumonia

We initially included 216 patients with lung cancer complicated with IP from three facilities. Of these, we excluded 103 patients due to the absence of IP (*n* = 13), the absence of FDG-PET/CT images (*n* = 17), history of chest or mediastinal radiation (*n* = 2), no chemotherapy (*n* = 75), and complication of other cancer (*n* = 2). In the remaining 113 cases, we conducted a statistical analysis of the factors that affect FDG accumulation in the interstitial lesions. The results showed that both TBR and TLR were significantly different between patients with “clinically diagnosed CTD-ILD” and those without CTD-ILD (Table 1). Therefore, 9 patients with clinically diagnosed CTD-ILD (6 without AE and 3 with AE) were excluded from the eligible patients, making a total of 104 patients for analysis (Fig. 3).

**Table 1:** Comparison of factors affecting FDG uptake of interstitial lesion

Factor	Patients without clinical CTD-ILD	Patients with clinical CTD-ILD	*p* value
*n*	104	9	
Contra TNR	3.42 (2.76, 4.48)	3.34 (2.75, 3.82)	0.584
Contra TBR	1.03 (0.86, 1.22)	1.27 (1.11, 1.41)	*0.041
Contra TLR	0.69 (0.58, 0.77)	0.86 (0.78, 0.96)	*0.013
Ipsi TNR	3.47 (1.63, 4.16)	0.95 (0.87, 1.03)	0.198
Ipsi TBR	1.04 (0.90, 1.33)	1.33 (1.12, 1.54)	*0.028
Ipsi TLR	0.71 (0.58, 0.84)	0.89 (0.77, 1.09)	*0.026

*CTD*-*ILD* connective tissue diseases associated interstitial lung disease, *Contra* contralateral, *IP* interstitial pneumonia, *Ipsi* ipsilateral, *TBR* target-to-blood ratio, *TLR* target-to-liver ratio, *TNR* target-to-normal ratio

^*^Statistically significant (*p* < 0.05)

The analysis included 104 patients with a median age of 72 years and 99% (103/104) had a smoking history (Table 2). The type of histology was small cell carcinoma (40.4%: 42/104), squamous cell carcinoma (27.9%: 29/104) and adenocarcinoma (23.1%: 24/104), and others (8.7%: 9/104). There were 17% of patients with postoperative recurrence. Although the types of systemic therapy varied, most patients received only cytotoxic agents, and only 13 patients (12.5%) were treated with at least one immune checkpoint inhibitor (ICI) in any line. The radiological pattern of IP was UIP (12.5%: 13/104), probable UIP (10.7%: 11/104), indeterminate for UIP (24.3%: 25/104) and Alternative (55.0%: 55/104) (Table 3). Honeycomb lung was present in 20.2% (21/104). The types of PET-CT devices and their usage are as follows: Discovery MI has 37 patients (35.6%), Biograph 64 has 35 patients (33.7%), Biograph 16 has 28 patients (26.9%), and GEMINI TF TOF 64 has 2 patients, and there is one patient each for Discovery 610 and Discovery ST.

**Table 2:** Characteristics of patients

	All (*N* = 104)	Without AE (*N* = 71)	With AE (*N* = 33)	*p* value
Backgrounds				
Median age (range)	72.0 (68.0, 76.0)	73.0 (68.5, 77.0)	71.0 (66.0, 74.0)	0.054
Gender (male/female)—no. (%)	81 (77.9)/23 (22.1)	55 (77.5)/16 (22.5)	26 (78.8)/7 (21.2)	1
Smoking history (former/never)—no. (%)	103 (99)/1 (1)	71 (100)/0 (0)	32 (97)/1 (3.0)	
PS (0–1/2–3/4)—no.(%)	96 (92.3)/7 (6.7)/1 (1.0)	65 (91.5)/5 (7.1)/1 (1.4)	31 (93.9)/2 (6.1)/0 (0.0)	0.006
Histology—no. (%)				0.392
Adenocarcinoma	24 (23.1)	19 (26.8)	5 (15.2)	
Squamous cell carcinoma	29 (27.9)	18 (25.4)	11 (33.3)	
Pleomorphic carcinoma	1 (1.0)	1 (1.4)	0 (0.0)	
Small cell carcinoma	42 (40.3)	28 (39.4)	14 (42.4)	
NOS	7 (6.7)	5 (7.0)	2 (6.1)	
Large cell neuroendocrine carcinoma	1 (1.0)	0 (0.0)	1 (3.0)	
Stage (ⅠA–ⅡB/Ⅲ A–C/Ⅳ A, B/recurrence)—no. (%)	5 (4.8)/29 (27.9)/51 (49.1)/19 (18.3)	5 (7.0)/20 (28.2)/32 (45.0)/14 (19.7)	0 (0.0)/9 (27.3)/19 (57.6)/5 (15.2)	0.588
Past surgical history	21 (20.2)	15 (21.1)	6 (18.2)	0.799
PD-L1 TPS (0–1%/1–49%/50–100%/unknown)	16 (17.0)/8 (8.5)/4 (4.4)/66 (70.2)	9 (14.5)/7 (11.3)/2 (3.2)/44 (71.0)	7 (21.9)/1 (3.1)/2 (6.2)/22 (68.8)	
Fine crackles	63(60.6)	41 (57.7)	22 (66.7)	0.723
Anti-fibrotic agents	1 (1.0)	1 (1.4)	0 (0.0)	
SpO_2_ (room air)	97.0 (96.0, 98.0)	97.0 (95.5, 97.5)	97.0 (96.0, 98.0)	0.267
Pulmonary function test				
FVC—no. (%)	95.2 (83.3, 107)	95.7 (80.7, 110)	95.0 (88.8, 14)	0.784
FEV1.0—no. (%)	76.0 (70.0, 79.1)	75.0 (69.6, 82.2)	76.5 (71.0, 79.0)	0.849
DLco—no. (%)	65.7 (52.5, 77.7)	62.0 (48,4 75.0)	70.6 (56.1, 78.4)	0.287
Examination data value (range)				
Alb (g/mL)	3.80 (3.50, 4.10)	3.80 (3.52, 4.00)	3.80 (3.40, 4.20)	0.605
CRP (mg/dL)	0.80 (0.20, 2.02)	0.90 (0.20, 2.25)	0.60 (0.20, 1.40)	0.475
Hb (g/dL)	13.3 (12.1, 14.2)	13.2 (12.0, 14.0)	13.4 (12.8, 14.4)	0.129
KL-6 (U/L)	583 (393, 840)	602 (377, 776)	560 (406, 861)	0.682
LDH (U/L)	225 (198, 272)	215 (196, 255)	240 (223, 340)	0.023
Regimens				
1 st line—no. (%)				0.124
CBDCA + (nab) PTX	53 (51.0)	39 (54.9)	14 (42.4)	
CBDCA (CDDP) + ETP	42 (40.3)	29 (40.9)	13 (39.4)	
CBDCA + ETP + ICI	2 (2.0)	0 (0.0)	2 (6.1)	
S-1	3 (2.9)	2 (2.8)	1 (3.0)	
Others	4 (3.8)	1 (1.4)	3 (9.1)	
2nd line—no. (%)	All (*N* = 83)	Without AE (*N* = 55)	With AE (*N* = 28)	0.535
S-1	28 (33.8)	18 (32.8)	10 (35.7)	
NGT	22 (26.5)	13 (23.6)	9 (32.1)	
CBDCA + ETP	14 (16.9)	11 (20.0)	3 (10.7)	
CBDCA + nabPTX	10 (12.0)	6 (10.9)	4 (14.3)	
Other	9 (10.8)	7 (12.7)	2 (7.2)	

*AE* acute exacerbation, *CBDCA* Carboplatin; *CDDP* Cisplatin, *DLco* diffusing capacity for carbon monoxide, *ETP* etoposide, *FEV1.0* forced expiratory volume in 1 s, *FVC* forced vital capacity, *ICI* immune checkpoint inhibitor, *nabPTX* nab-paclitaxel, *PS* performance status; *PTX* Paclitaxel; *S-1* Tegafur Gimeracil Oteracil Potassium

**Table 3:** Imaging patterns between those with and without AE

	All (*N* = 104)	Without AE (*N* = 71)	With AE (*N* = 33)	*p *value
UIP—no. (%)	13 (12.5)	8 (11.3)	5 (15.2)	0.887
Probable UIP—no. (%)	11 (10.7)	7 (9.8)	4 (12.5)	
Indeterminate for UIP—no. (%)	25 (24.3)	18 (25.3)	7 (18.8)	
Alternative diagnosis—no. (%)	55 (52.9)	38 (53.5)	17 (51.5)	
Honeycomb lung—no. (%)	21 (20.2)	15 (21.1)	6 (18.2)	

*AE* acute exacerbation, *HRCT* high-resolution computed tomography, *IP* interstitial pneumonia, *UIP* usual IP

### Risk factors for AE

**Fig. 4:**
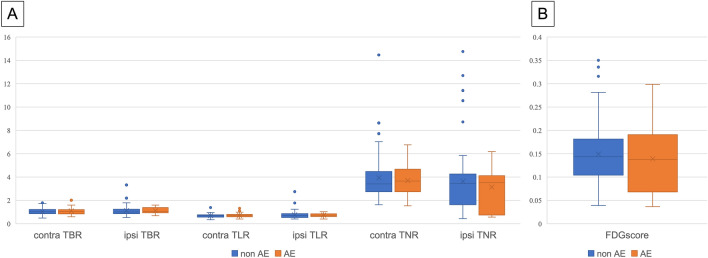
FDG accumulation of interstitial lesions with and without AE. *AE* acute exacerbation, *Contra* contralateral, *Ipsi* ipsilateral, *TBR* target-to-blood ratio, *TLR* target-to-liver ratio, *TNR* target-to-normal ratio

The incidence of AE was 31.7% (33/104) for all grades and 18.3% (19/104) for grade 3 or higher. The radiological patterns of AE were HP-like in 13 patients (36.7%), NSIP in 10 patients (33.4%), COP in 5 patients (16.7%), AIP in 1 patient (3.3%), and 4 patients had no CT on AE (Supplement Table 1). Although the types of systemic therapy that caused AE varied, most patients (29/33, 87.9%) received only cytotoxic agents, while a small number (4/33, 12.1%) were treated with ICI either alone or in combination regimens (Supplementary Table 2). There were no significant differences without PS and LDH in patient characteristics, imaging patterns between those with and without AE (Tables 2 and 3). In addition, there was no difference in SUVpeak and FDGscore for each imaging pattern (Supplement Table 3). SUVpeak in the ipsilateral and contralateral IP to the reference uptake, and FDGscore did not differ between those with or without AE (Fig. 4). Univariate analysis also showed no significant difference in FDG accumulation with or without AE, and no significant risk factors were identified (Table 3). The results were also similar when comparing Grade 1, 2 to Grade 3 or higher in cases of AE (Supplement Table 4).

**Table 4:** Univariate logistic regression analysis of clinical and imaging factor

	Odds ratio	95% CI	*p* value
Contra TNR	1.910	0.463–7.91	0.370
Contra TBR	2.240	0.303–16.5	0.430
Contra TLR	0.914	0.702–1.19	0.504
Ipsi TNR	0.926	0.771–1.11	0.414
Ipsi TBR	1.00	0.322–3.12	0.997
Ipsi TLR	0.835	0.185–3.79	0.815
FDG score	0.0903	0.000–54.1	0.461
Age	0.935	0.868–1.01	0.076
Alb	1.249	0.494–3.12	0.645
CRP (mg/dL)	1.010	0.920–1.11	0.833
DLco < 80%	0.792	0.125–5.01	0.804
FVC > 80%	2.170	0.636–7.38	0.216
Hb	0.993	0.962–1.02	0.633
KL-6 (U/L)	1.00	0.999–1.00	0.988
Honeycomb lung	0.830	0.290–2.38	0.728
LDH (U/L)	1.000	0.999–1.01	0.202
Past surgical history	0.830	0.290–2.38	0.728

*AE* acute exacerbation, *Alb* albumin, *CRP* C-reactive protein, *DLco* diffusing capacity for carbon monoxide, *FVC* forced vital capacity, *Hb* hemoglobin, *LDH* lactate dehydrogenase, *TBR* target-to-blood ratio, *TLR* target-to-liver ratio, *TNR* target-to-normal ratio

## Discussion

To our knowledge, the present study is the largest multi-institutional research investigating the correlation between FDG uptake and the incidence of AE in patients with lung cancer complicated with IP. In the present study, no association was observed between FDG accumulation in the interstitial lesions and the incidence of AE of IP after systemic therapy. Previous reports have noted that the intensity of accumulation in the background lung of idiopathic pulmonary fibrosis is associated with the prognosis of IP and the tendency of AE [11]. Regarding IP-complicated lung cancer, according to the study by Akaike et al., the AE group showed a higher accumulation of FDG than the non-AE group, but the difference was not significant in patients with ILD [15]. This result was supported by the study of Yamazaki et al. reporting that lung cancer patients with and without IP receiving immune checkpoint inhibitors with higher SUV values (indicating more active IP) in the noncancerous lung (NCL) had a significantly higher incidence of AE [18]. However, previous studies had small sample sizes [15, 18], and the difference of FDG accumulation could have been inconsistent. In contrast, this study was a multicenter study and analyzed a relatively large sample size with IP patients only. Thus, the result was believed to be more accurately evaluated.

In this study, several possible reasons may explain why no association was observed between FDG accumulation in interstitial lesions and the incidence of AE. First, the relationship between the mechanisms of systemic therapy-related AE and FDG uptake may have influenced the results. In typical IP, FDG accumulation is considered to reflect inflammatory and fibrotic activity [19, 20] and may be associated with the risk of AE [11]. However, systemic therapy-related AEs are believed to occur via two main pathways: cytotoxic and immune/allergic mechanisms [21, 22]. The cytotoxic anticancer agents that were predominantly used in this study are generally associated with the former. This mechanism may differ from that of AEs observed in typical IP. Therefore, in AEs caused by cytotoxic agents, underlying lung conditions with high inflammatory and fibrotic activity may not necessarily serve as risk factors. Moreover, there was considerable variation in the types of anticancer agents used, and the risk of AE may differ depending on the specific agent. Therefore, in addition to patient-related factors, the intrinsic potential of each drug to induce AE may have influenced the results. Owing to the diversity of agents used, we were unable to examine whether differences in FDG uptake or imaging patterns affected the frequency of AE among patients receiving the same drug. Second, the relationship between fibrosis and FDG accumulation may have influenced the results. Previous reports on the risk of AEs with cytotoxic agents have identified factors such as the UIP pattern [23, 24], extent of fibrosis [25], and reduced lung volume [24–27]. These findings suggest that lungs with advanced fibrosis and diminished reserve capacity for cytotoxic lung damage are more susceptible to chemotherapy-related AE. In such fibrotic lungs, FDG uptake is not always elevated, which may partly explain the lack of correlation between FDG accumulation and the frequency of AE observed in this study. In addition, many cases had only subtle radiographic findings, making it difficult to accurately classify the imaging patterns. Such cases may progress to a UIP pattern in the future, even if the current radiographic findings are subtle. However, at this stage, a large number of cases were categorized as having “alternative” or “indeterminate” patterns. This may have also influenced the results, where the frequency of AE was not higher in patients with a UIP pattern in this study. Owing to the small number of patients clearly diagnosed with the UIP pattern, it was not feasible to compare FDG uptake, the degree of fibrosis progression, and AE frequency specifically within the UIP subgroup in this study.

Third, differences in the methods used to assess the extent and degree of FDG uptake may have influenced the results. In a previous report, Yamazaki et al. used AI to investigate the relationship between FDG accumulation in NCL of lung cancer and the onset of ICI-ILD, and showed that high uptake of FDG in NCL is associated with the onset of ICI-ILD and may be helpful as a risk stratification tool before ICI therapy [18]. In contrast, FDG uptake in our study was assessed using localized visual evaluation and a simplified scoring system to estimate the extent and intensity of inflammation in the lungs. The assessments of the two evaluators were generally consistent, with a concordance rate of 76.9%, allowing a 5% margin of error. However, no significant differences in FDG uptake were observed between the groups. One reason for the discrepancy between our results and those of Yamazaki et al. may be that our study included very few patients treated with ICIs, suggesting a different mechanism of chemotherapy-related AE, as discussed above. In addition, the lack of AI-based analysis in our study might have affected the results. Although localized visual assessment remains a simple and widely used method in routine clinical practice and in previous reports, AI-based image segmentation is not yet common and requires considerable time for analysis. Furthermore, in the evaluation of FDG uptake in IP-complicated lung cancer, it is difficult to distinguish adjacent organs, such as the liver, heart, and spleen, located directly below the pleura, as well as lung cancer lesions. This poses a problem in terms of accuracy, as it is difficult to automate, and important information may be missed. Nevertheless, in the future, quantitative assessment of FDG uptake across the entire background lung using AI or other advanced techniques may yield different results.

This study had some limitations. First, although the overall sample size was sufficient compared to that in previous reports, the number of patients with specific backgrounds, such as those classified by chemotherapeutic agent type or those with a UIP pattern, was small, resulting in an insufficient sample size. This precluded a more detailed analysis of risk factors based on imaging patterns and patient characteristics. In addition, previous studies often used SUVmax or SUVmean directly for analysis. However, this method does not account for differences in sensitivity between PET-CT devices. Therefore, SUVpeak should be corrected to eliminate facility differences to standardize the method. In our study, we tried to adjust SUVpeak values of all PET-CT device based on references such as blood pools and the liver. However, such corrections do not exactly eliminate the differences between devices.

## Conclusions

Among lung cancer patients complicated with IP, FDG accumulation in the interstitial lesion area was not significantly causally related to the occurrence of drug-induced AE during chemotherapy in this multicenter study. Therefore, the strength and extent of FDG accumulation do not always correlate with the risk of AE, and even cases with or without FDG accumulation should be watched for AE.

## Supplementary Information

Below is the link to the electronic supplementary material.

Supplementary file1 (DOCX 18 KB)
