# Supplementary material for: Association between FDG accumulation in interstitial lesions and acute exacerbation risk in lung cancer: multicenter analysis
Source: Jpn J Radiol. 2025 Sep 12;44(1):147–55. doi: 10.1007/s11604-025-01869-4 (PMC12769495; doi:10.1007/s11604-025-01869-4)
Supplement: Supplementary file 1 — Supplementary file1 (DOCX 18 KB) [file 11604_2025_1869_MOESM1_ESM.docx]

**Supplement 1 The radiological patterns of AE**

| type of AE | n=33 |
| --- | --- |
| AIP/DAD-like pattern -no.(%) | 1 (3.3) |
| COP like pattern -no.(%) | 5 (16.7) |
| HP like pattern -no.(%) | 13 (36.7) |
| NSIP like pattern -no.(%) | 10(33.4) |
| no CT -no.(%) | 4 (10.0) |

Abbreviations: AE, acute exacerbation; AIP, acute interstitial pneumonia; COP, cryptogenic organizing pneumonia; DAD, diffuse alveolar damage; HP, hypersensitivity pneumonia; NSIP, nonspecific interstitial pneumonia

**Supplement 2 The Regimen of anticancer drugs at the onset of AE**

| **Kind of regimen resulting in AE** | n=33 |
| --- | --- |
| Atezolizumab-no.(%) | 2 ( 6.7) |
| CBDCA+ETP-no.(%) | 3 (10.0) |
| CBDCA+ETP+Atezolizumab-no.(%) | 1 ( 3.3) |
| CBDCA+ETP+Durvalumab-no.(%) | 1 ( 3.3) |
| CBDCA+nabPTX-no.(%) | 2 ( 6.7) |
| CBDCA+S-1-no.(%) | 1 ( 3.3) |
| DOC+RAM-no.(%) | 1 ( 3.3) |
| nabPTX-no.(%) | 1 ( 3.3) |
| NGT-no.(%) | 9 (30.0) |
| S-1-no.(%) | 4 (13.3) |
| VNR-no.(%) | 5 (16.7) |

Abbreviations: AE, acute exacerbation; CBDCA, Carboplatin；CDDP, Cisplatin; DOC,Docetaxel; ETP, etoposide; nabPTX, nab-paclitaxel; NGT, Nogitecan; PTX, Paclitaxel；RAM, Ramucirmab; S-1, Tegafur Gimeracil Oteracil Potassium;

**Supplement 3 SUVpeak for each imaging pattern**

| **Factor** | alternative | indeterminate for UIP | probable UIP | UIP | p value |
| --- | --- | --- | --- | --- | --- |
| **n** | 55 | 25 | 11 | 13 |  |
| contra TNR | 3.42 (2.70, 4.62) | 3.74 (2.88, 4.31) | 3.78 (2.81, 4.47) | 3.33 (2.77, 4.09) | 0.98 |
| contra TBR | 1.03 (0.88, 1.25) | 1.02 (0.83, 1.14) | 1.03 (0.91, 1.23) | 1.06 (0.85, 1.11) | 0.912 |
| contra TLR | 0.70 (0.58, 0.78) | 0.64 (0.56, 0.74) | 0.65 (0.47, 0.78) | 0.69 (0.60, 0.77) | 0.581 |
| ipsi TNR | 3.67 (2.04, 4.71) | 3.45 (0.95, 3.81) | 3.58 (3.36, 4.38) | 2.76 (0.79, 3.44) | 0.479 |
| ipsi TBR | 1.07 (0.90, 1.39) | 1.01 (0.91, 1.16) | 1.08 (0.94, 1.29) | 1.10 (0.80, 1.25) | 0.828 |
| ipsi TLR | 0.73 (0.61, 0.91) | 0.66 (0.58, 0.78) | 0.71 (0.49, 0.76) | 0.74 (0.56, 0.79) | 0.559 |
| FDGscore | 0.13 (0.09, 0.17) | 0.14 (0.08, 0.19) | 0.14 (0.11, 0.16) | 0.16 (0.13, 0.26) | 0.264 |

Abbreviations: IP, interstitial pneumonia; SUV, Standardized Uptake Value; TBR, target-to-blood ratio; TLR target-to-liver ratio; TNR, target-to-normal ratio

Supplement 4　**SUVpeak comparing Grade 1,2 to Grade 3 or higher in cases of AE.**

|  | All (N=36) | Grade 1,2 (N=17) | Grade3 over (N=19) | p value |
| --- | --- | --- | --- | --- |
| contra TNR | 3.65 (2.77, 4.64) | 3.18 (2.82, 4.36) | 3.70 (2.76, 4.71) | 0.943 |
| contra TBR | 1.05 (0.85, 1.18) | 1.02 (0.83, 1.16) | 1.05 (0.96, 1.23) | 0.54 |
| contra TLR | 0.69 (0.61, 0.78) | 0.67 (0.56, 0.71) | 0.75 (0.62, 0.79) | 0.264 |
| ipsi TNR | 3.53 (1.10, 4.00) | 3.74 (3.00, 4.37) | 3.28 (0.75, 3.96) | 0.443 |
| ipsi TBR | 1.06 (0.95, 1.38) | 1.07 (0.99, 1.39) | 1.03 (0.89, 1.26) | 0.372 |
| ipsi TLR | 0.73 (0.61, 0.81) | 0.75 (0.62, 0.88) | 0.70 (0.61, 0.76) | 0.351 |
| FDGscore | 0.14 (0.10, 0.18) | 0.14 (0.10, 0.18) | 0.17 (0.09, 0.22) | 0.394 |

Abbreviations: AE, acute exacerbation; IP, interstitial pneumonia; SUV, Standardized Uptake Value; TBR, target-to-blood ratio; TLR target-to-liver ratio; TNR, target-to-normal ratio
